# Bidirectional Transcription Directs Both Transcriptional Gene Activation and Suppression in Human Cells

**DOI:** 10.1371/journal.pgen.1000258

**Published:** 2008-11-14

**Authors:** Kevin V. Morris, Sharon Santoso, Anne-Marie Turner, Chiara Pastori, Peter G. Hawkins

**Affiliations:** 1 Department of Molecular and Experimental Medicine, The Scripps Research Institute, La Jolla, California, United States of America; 2 The Kellogg School of Science and Technology, The Scripps Research Institute, La Jolla, California, United States of America; 3 Oncology Institute of Southern Switzerland, Bellinzona, Switzerland; University of California San Francisco, United States of America

## Abstract

Small RNAs targeted to gene promoters in human cells have been shown to modulate both transcriptional gene suppression and activation. However, the mechanism involved in transcriptional activation has remained poorly defined, and an endogenous RNA trigger for transcriptional gene silencing has yet to be identified. Described here is an explanation for siRNA-directed transcriptional gene activation, as well as a role for non-coding antisense RNAs as effector molecules driving transcriptional gene silencing. Transcriptional activation of p21 gene expression was determined to be the result of Argonaute 2–dependent, post-transcriptional silencing of a p21-specific antisense transcript, which functions in Argonaute 1–mediated transcriptional control of p21 mRNA expression. The data presented here suggest that in human cells, bidirectional transcription is an endogenous gene regulatory mechanism whereby an antisense RNA directs epigenetic regulatory complexes to a sense promoter, resulting in RNA-directed epigenetic gene regulation. The observations presented here support the notion that epigenetic silencing of tumor suppressor genes, such as p21, may be the result of an imbalance in bidirectional transcription levels. This imbalance allows the unchecked antisense RNA to direct silent state epigenetic marks to the sense promoter, resulting in stable transcriptional gene silencing.

## Author Summary

Non-coding RNAs have been shown to modulate transcriptional expression of genes in human cells. This form of gene regulation has been shown to be the result of RNA directing silent state epigenetic changes to the targeted gene promoter. Shortly after this seminal observation, small RNAs targeted to AT-rich regions of gene promoters were shown to modulate gene activation, termed RNA activation. While much is known regarding non-coding RNA-mediated transcriptional gene silencing, the mechanism of RNA activation has remained elusive. Here, we present evidence that RNA activation is the result of deregulation of endogenous bidirectional transcription. The antisense transcript in the bidirectionally transcribed gene is shown here to be operative in directing silent state epigenetic marks to the sense gene promoter. Suppression of the antisense transcript results in gene activation. Overall, these data support the notion that bidirectional transcription is an endogenous mechanism whereby RNA-directed gene regulation is operative and that RNA activation is the result of a disruption of this endogenous pathway. An understanding of this mode of RNA-based regulation will prove exceptionally useful in understanding gene expression as well as potential therapeutic approaches to controlling gene expression in human cells.

## Introduction

Over the past few years it has become increasingly apparent that many RNA-mediated modes of gene regulation are operative in biological systems [1]. It is only now becoming appreciated just how pervasive this network is and to what extent it may be possible to apply this phenomenon for therapeutic benefit. Adding to the complexity of this regulatory network is the recent observation that small non-coding RNA mediated transcriptional regulation can act in both a suppressive [2],[3] and activating manner [4],[5] in human cells. Mechanistically, small RNA directed transcriptional gene suppression functions by specific targeting of epigenetic modifications to gene promoters [6],[7]. This activity requires Argonaute 1 (Ago-1)[8],[9] and a low-copy promoter associated RNA (pRNA) spanning the targeted loci [6]. While much is known regarding the mechanism of small RNA directed transcriptional gene silencing in human cells [10], little is known regarding the identity of possible endogenous triggers which may drive this pathway, or how small RNAs can also direct gene activation.

Small RNA directed transcriptional activation has been reported in human cells for p21, E-cadherin [5],[11], and the progesterone receptor (PR)[4],[12]. Importantly, these small RNAs were designed to target AT rich regions of gene promoters, required the activity of the 5′ end of the siRNA, and Argonaute 2 (Ago-2) [5],[11]. RNA activation has also been observed with small RNAs targeted to the HIV-1 LTR/promoter. However, the observed gene activation appeared to be the result of an indiscriminate off-target effect [13].

## Results

During the course of studies to determine the mechanism involved in small RNA directed gene activation, we determined that the gene activating siRNA p21-322 [5] could, in theory, bind to and potentially target a p21 antisense transcript (Figure S1A). A similar observation was noted for the E-cadherin targeted siRNA E-cad640 and miRNA miR373, both of which had been shown to mediate gene activation of E-cadherin (Figure S2A) [5],[11]. In addition to being susceptible to siRNA mediated gene activation [5],[11], both the p21 and E-cadherin genes have been shown to exhibit bidirectional transcription [14] and an antisense RNA has been implicated in siRNA directed gene activation of progresterone [12].

**Figure 1:**
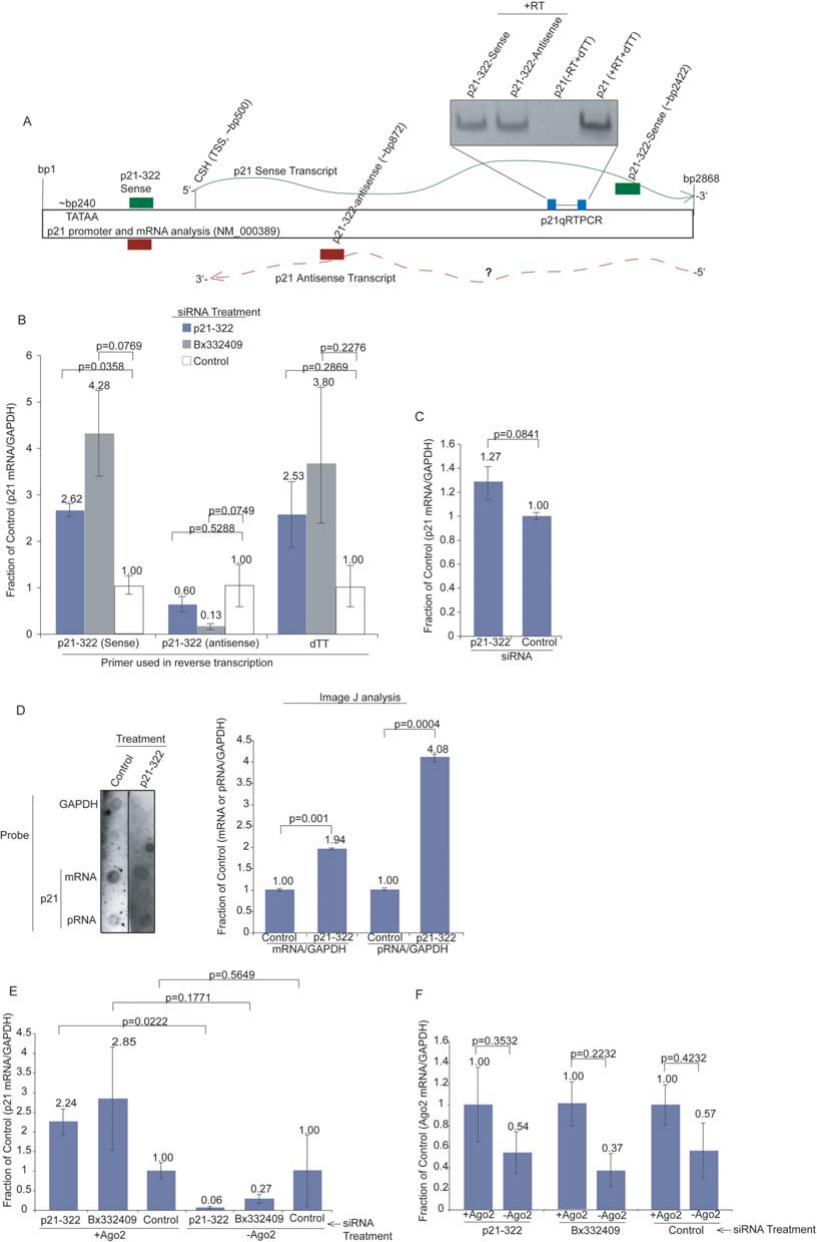
siRNA Targeting of the p21 Antisense Transcripts Results in Gene Activation of p21. (A) The p21-322 siRNA sequence can specifically reverse transcribe both sense and antisense p21 transcripts. The p21 promoter and mRNA are shown schematically. The transcriptional start site (TSS), p21-322 siRNA target site [5], and other regions where the p21-322 sense or p21-322 antisense strands can hybridize are shown. The hybridization of p21-322 sense or antisense was confirmed by primer specific cDNA conversion followed by RT-PCR specifically for p21 mRNA expression (inset). (B) Targeted suppression of the p21 antisense RNA, EST Bx332409 [14], results in pronounced p21 sense/mRNA expression. MCF-7 cells were transfected with siRNAs, p21-322, Bx332409, or R854 (Control) [3] and contrasted 48 hrs later. The averages of triplicate transfected cultures pooled together are shown with the standard deviations based on qRT-PCR analysis performed in triplicate for each sample along with the p values from paired T tests. (C) Suppression of antisense transcription results in an increase in RNAPII sense/mRNA transcriptional activity. Nuclear run-ons analysis measured by quantitative RT-PCR are shown from duplicate treated cultures with the respective ranges and p values from a paired T test. (D) Both the mRNA and pRNAs are increased in transcriptional expression at the p21 promoter following p21-322 treatment. Nuclear run-on data from (C) was assessed by dot blot analysis. Nuclear run-ons are shown from single treated cultures with the averages determined using Image J analysis standardized to GAPDH and the standard deviations shown with p values from a paired T-test. (E) Gene activation resulting from siRNA targeting of the p21 antisense RNA requires Ago-2. MCF-7 cells were transfected first with either shRNA expressing plasmid targeted to Ago-2 [23](-Ago2) or a control plasmid (+Ago2) followed by a second transfection with siRNAs, p21-322, Bx332409, or R854 (Control) [3]. Forty-eight hours later p21 mRNA expression was determined relative to GAPDH. The averages from triplicate transfected cultures are shown with the standard deviations and p values from the respective paired T tests. (F) Ago-2 expression levels in the p21 antisense RNA silenced cultures. Ago-2 mRNA expression was determined in the p21 antisense siRNA targeted cultures (E) 48 hrs post-transfection relative to GAPDH expression. The averages of triplicate transfected cultures are shown with the standard deviations and results from a paired T test.

To determine the ability of the p21 activating siRNA, p21-322, to interact with either a p21 sense or antisense transcript, directional reverse transcription was performed. Both strands of the p21 siRNA p21-322 (Table S1 and [5]) effectively primed reverse transcription of the p21 sense or antisense transcripts (Figure 1A). Upon closer examination of the reported ESTs known to be antisense to p21 [14], it was determined that one particular EST, Bx332409, contained significant homology to the gene activating p21-322 siRNA [5]. Similar to observations with p21, both E-cadherin 640 and miR373, shown previously to function as activating RNAs [5],[11], were also capable of priming the reverse transcription of E-cadherin (Figures S2B and S2C, respectively). E-cadherin has also been reported to contain several antisense ESTs (Figure S2A and [14]).

Suppression of the p21 antisense RNA Bx332409 with the siRNA si-Bx332409, which is specifically targeted to the locus partially targeted by p21-322 (Figure S1A), demonstrated a significant suppression of the p21 antisense transcript which correlated with a distinct increase in p21 sense/mRNA transcript expression (Figures 1B and S1B). Similar to observations in p21, cultures treated with E-cadherin 640 or miR373 demonstrated a significant suppression of the E-cadherin antisense transcription which correlated with a distinct increase in E-cadherin sense/mRNA transcript expression (Figure S2D). The observed increase in p21 mRNA expression appeared to be the result of increased transcriptional activity in the p21 promoter as determined by nuclear run on analysis (Figures 1C and 1D). Similar to previous observations, Argonaute 2 (Ago-2) appeared to be required for siRNA p21-322 and Bx332409 mediated gene activation (Figures 1E and 1F)[4],[5],[11]. These data suggest that post-transcriptional silencing of the p21 bidirectional transcript EST Bx332409 is sufficient for modulating an increase in p21 sense strand transcription.

**Figure 2:**
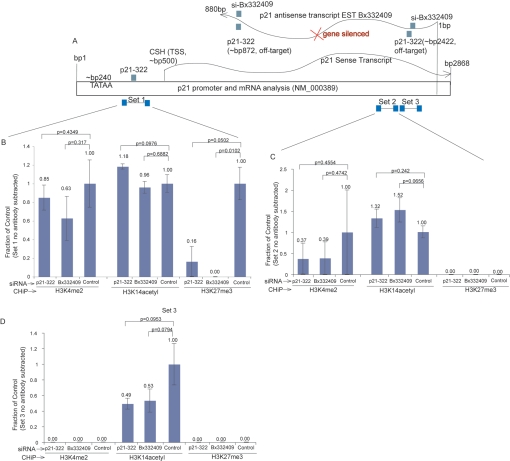
Suppression of p21 antisense transcription results in a loss of suppressive H3K27me3 epigenetic mark at the p21 promoter. The activating marks (H3K4 and H3K14) and the suppressive mark H3K27me3 were assessed by ChIP analysis in p21-322 and Bx332409 siRNA treated cells. (A) Schematic of the various p21-322 and Bx33409 siRNA target loci as well as primer sets (sets 1–3) used in the subsequent ChIP analysis are shown. The results from duplicate siRNA transfected cultures are shown with primer sets spanning (B) the p21-322 promoter target site (Set A), (C) in the coding region of p21, and (D) spanning the p21-322 and Bx332409 targeted site in the p21 mRNA. The standard error of the means is shown for quadruplicate measurements of the duplicate transfected cultures and the respective p values from paired T tests.

Small non-coding RNA directed transcriptional gene silencing of gene promoters correlates with the initial recruitment of Ago-1, followed shortly thereafter by silent state epigenetic marks such as histone 3 lysine 27 tri-methylation (H3K27me3) at the targeted locus (reviewed in [10]). Recently, bidirectionally transcribed genes have been shown to modulate epigenetic modifications that can function in tumor suppressor gene silencing [14]. Thus, we speculated that the endogenous function for the p21 antisense RNA Bx332409 is to direct epigenetic modifications which regulate p21 sense/mRNA transcription. To test this supposition, chromatin immunoprecipitation assays (ChIP) were performed for activating (H3K4me2, AcH3K14) and suppressive (H3K27me3) epigenetic modifications at various loci in the p21 gene following siRNA directed gene activation (Figure 2A). Following suppression of the antisense transcript, a loss of H3K27me3 was observed at the p21 promoter (Figure 2B).

In contrast, no significant changes in H3K27me3 were observed within the p21 coding region (Figure 2C) or at the si-Bx332409 target site of the p21 coding region (Figure 2D). While there were changes in H3K14me2 at both the siBx332409 site and within the p21 coding region it is difficult to interpret a trend. Overall, these data indicate that the suppression of the p21 antisense transcript EST Bx332409 results in a loss of the suppressive H3K27me3 mark at the p21 sense promoter.

**Figure 3:**
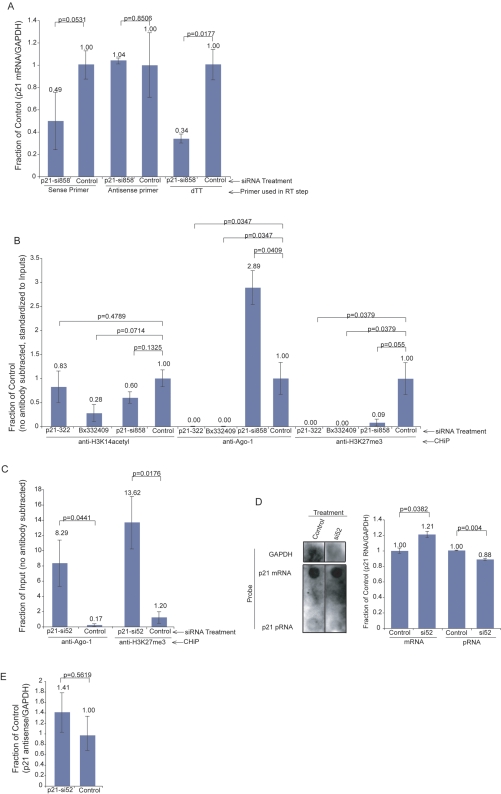
Suppression of p21 sense/mRNA expression results in the recruitment of Ago-1 and H3K27me3 to the p21 promoter. (A) siRNA p21-si858, which is targeted to the p21 sense/mRNA, suppresses p21 sense/mRNA transcription with no effect on p21 antisense transcription. Results from triplicate transfected MCF-7 cell cultures are shown with the standard deviations and p values from paired T tests. (B) The suppression of p21 sense transcription results in an increase, 24 hrs post-siRNA transfection, in Ago-1 at the p21 promoter whereas suppression of the p21 antisense transcript results in a loss of Ago-1 enrichment at the p21 promoter. Triplicate measurements from a single ChIP assay are shown with the standard deviations and p values from paired T tests. (C) The suppression of p21 sense transcription results in an enrichment of Ago-1 and silent state epigenetic marks H3K27me3, specifically at the p21 promoter 48 hrs post-siRNA treatment. The averages with the standard error of the means are shown from duplicate transfected cultures and p values from paired T tests. (D) Suppression of p21 sense/mRNA transcription results in an increase in transcription at the p21 promoter loci as determined by nuclear run on analysis. A dot blot and Image J analysis is shown from 2 separate experiments pooled together. The averages, standard deviations, and p values from paired T tests are also shown from the Image J analysis. (E) Suppression of p21 sense/mRNA transcription results in an increase in RNAPII p21 antisense transcriptional activity. Nuclear run-on analysis measured by quantitative RTPCR are shown with the averages from 2 separate experiments with duplicate treated cultures assessed spanning 24–48 hrs post-siRNA treatment. The standard error of means shown along with p values from paired T tests.

Our previous observations suggest that the p21 antisense transcript Bx332409 functions to negatively regulate p21 (sense, mRNA) expression. As such, we speculated that the suppression of the p21 sense transcript would facilitate an imbalance whereby the antisense, unimpeded by the sense transcript, might modulate epigenetic changes at the p21 sense promoter. To explore this concept, siRNAs were screened for suppression of p21 sense/mRNA expression. Two siRNAs were shown to significantly suppress p21 mRNA expression, si52 and si858 (Figure S3). Interestingly, suppression of the p21 sense transcript did not noticeably affect levels of the p21 antisense transcripts in the coding region of p21 (Figure 3A). However, when the p21 promoter was assessed for local changes in the epigenetic marks 24 hours following the suppression of p21 sense/mRNA, a noticeable increase in Ago-1 and to a lesser extent the suppressive histone mark H3K27me3 was observed (Figure 3B). A more pronounced enrichment of both Ago-1 and H3K27me3 was observed 48 hours following suppression of p21 sense/mRNA expression (Figure 3C). Moreover, nuclear run-on analysis indicated an increase in transcription (Figure 3D) that was predominantly antisense (Figure 3E), thus supporting the notion that the observed epigenetic changes in the p21 sense/mRNA promoter might be the result of antisense RNA mediated control of gene transcription. Overall, these data suggest that the p21 antisense RNA is operative in directing the recruitment of Ago-1 to the p21 gene promoter as well as the emergence of increased silent state epigenetic marks. This observation is strikingly similar to previous observations of siRNA directed transcriptional gene silencing [10] and suggestive of a role for non-coding RNAs as the endogenous effector molecule(s) driving transcriptional gene silencing in human cells.

**Figure 4:**
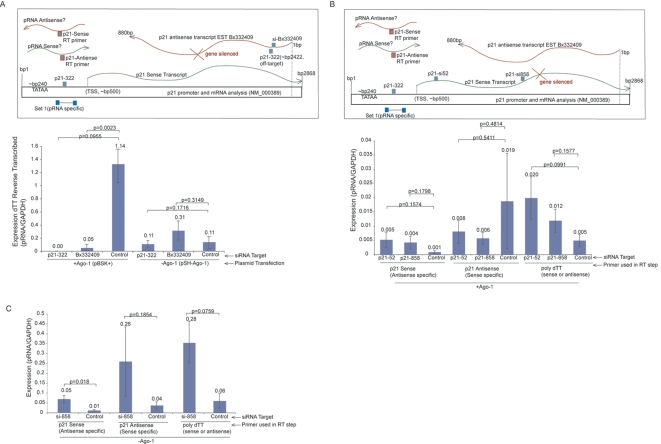
Both the p21 antisense transcript and Ago-1 are required for the regulation of p21 promoter-associated RNAs and the suppression of promoter activity. (A) The antisense transcript, Bx332409, was silenced post-transcriptionally (shown in inset) with p21-322 or si-Bx332409 siRNAs (50 nM) in the presence or absence of Ago-1. In the absence of Ago-1, an enrichment of dTT reverse transcribed p21 promoter associated RNAs (pRNA) are observed. Averages from triplicate transfected cultures, standard deviations, and p values from paired T tests are shown. (B and C) The p21 sense transcript, coding mRNA, was silenced post-transcriptionally (shown in inset) with p21-52 or p21-858 siRNAs (50 nM) in the presence (B) or absence (C) of Ago-1. In the presence of Ago-1 and p21 antisense transcription (B) an increase in p21 antisense pRNAs are observed with a concomitant loss of p21 sense pRNAs. In the absence of Ago-1 however (C) a loss of antisense pRNAs and an enrichment for sense transcribed p21 pRNAs is observed. Averages, standard deviations, and p values from paired T tests are shown from triplicate transfected cultures.

Small RNA directed transcriptional gene silencing (TGS) has been shown to require a low-copy promoter associated RNA (pRNA) spanning the targeted locus in the gene promoter [6]. Transcriptional activity within the p21 promoter was shown to be down regulated when the p21 antisense RNA was suppressed in an Ago-1 dependent manner (Figures 4A and S4). Conversely, when the p21 sense/mRNA was suppressed, an increase in p21 pRNA transcription was observed which consisted predominantly of antisense stranded transcripts (Figure 4B). However, when both the p21 sense/mRNA and Ago-1 were suppressed, there was a loss in the enrichment of antisense p21 pRNA and a notable increase in sense stranded p21 pRNAs (Figure 4C). These data suggest that antisense transcription spanning the p21 promoter, along with Ago-1, are involved in the regulation of p21 sense/mRNA transcriptional expression. Taken together these data suggest that the balance between p21 sense and antisense bidirectional transcription is maintained by the action of p21 antisense RNA directed epigenetic modifications that endogenously regulate RNAPII activity at the p21 promoter.

## Discussion

Clearly, a far greater amount of the human genome is transcribed in both the sense and antisense direction than had previously been envisioned [12],[15],[16],[17],[18]. Exactly what roles bidirectional transcription may play in the cell cycle, and how it therein operates is not clear. Interestingly, several bidirectionally transcribed genes contain significant overlap with their complimentary RNA counterpart in the 3′ UTR of the respective transcripts [18]. This observation, juxtaposed with the observation that the majority of micro RNAs (miRNAs) target 3′UTRs of genes [19], might be suggestive of a possible role for miRNAs in regulation of bidirectional transcription and non-coding RNAs.

**Figure 5:**
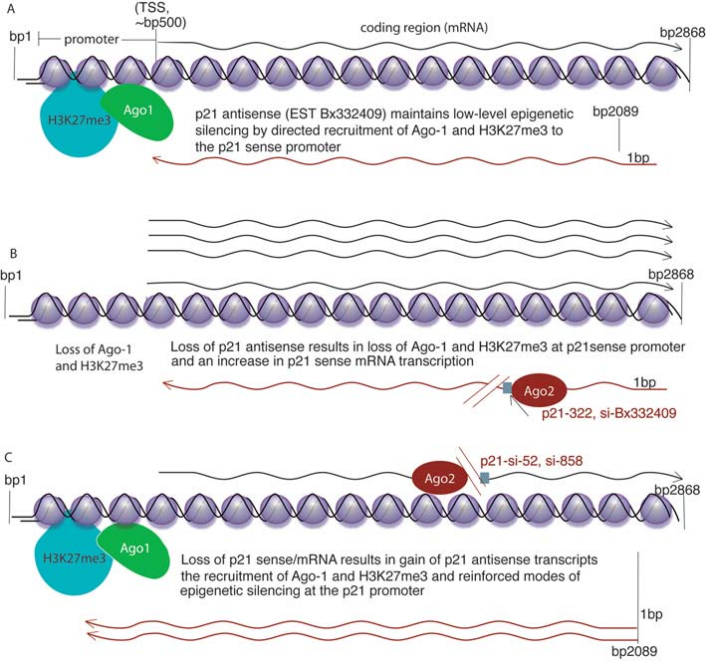
Model for bidirectional RNA mediated transcriptional regulation of gene expression in human cells. (A) Bidirectional transcription is presumed to exert a controlled equilibrium of endogenous p21 gene expression, whereby the antisense transcript directs Ago-1 dependent low-level epigenetic regulation, H3K27me3, at the p21 sense/mRNA promoter. (B) Upon suppression of the antisense transcript the directed H3K27me3 is diminished, allowing for enhanced p21 sense/mRNA transcriptional gene activation. (C) When p21 sense/mRNA expression is suppressed, a release of regulatory pressure is exerted on the p21 antisense transcript allowing for p21 antisense directed Ago-1 and H3K27me3 enrichment at the p21 sense/mRNA promoter. Ago-1 has been observed to be required for the early stages of RNA directed transcriptional gene silencing [9], thus the early recruitment of Ago-1 to the p21 sense promoter may function as a re-enforcing loop to exert stable epigenetic silencing on p21 gene expression.

Recent examples of bidirectional transcription have demonstrated a role for these RNAs in the epigenetic regulation of gene expression [14],[20],[21]. We show here that siRNA mediated gene activation of p21 is not the result of direct promoter targeting, but rather is operative via post-transcriptional suppression of a p21 antisense RNA, EST Bx332409. The data presented here are suggestive of a model for bidirectional gene regulation in human cells (Figure 5). At steady state, endogenous p21 contains comparable levels of both sense and antisense transcripts (Figure 5A and Table S2). When a reduction in p21 antisense transcription occurs, there is a loss of the low-level antisense directed H3K27me3 suppressive mark at the p21 sense promoter and an increase in p21 sense/mRNA expression (Figure 5B). This observation suggests that H3K27me3 is enriched, to some extent, at the p21 promoter by the action of the p21 antisense transcript, Bx332409, via the mechanism utilized by siRNAs or small antisense RNAs to direct transcriptional gene silencing [10]. Conversely, a decrease in p21 sense/mRNA expression results in p21 antisense mediated Ago-1 recruitment to the p21 sense/mRNA promoter (Figure 5C), followed shortly thereafter with an enrichment of H3K27me3, similar to the observed mechanism whereby siRNAs direct transcriptional gene silencing [10]. The inverse situation whereby the sense transcript can regulate the antisense may also be functional, though no data supporting this has been presented. Regardless, such a molecular pathway, where an imbalance in bidirectional transcription leads to dominant antisense RNA expression, resulting in directed transcriptional gene silencing of the sense promoter, might explain observations of epigenetic silencing of tumor suppressor genes, such as p21, in several human cell cancers [22].

## Materials and Methods

### p21siRNA, E-cadherin 640, and miR373 Transfections and mRNA Knockdown

All of the siRNA used in this study (Table S1) were generated using the Silencer siRNA construction kit (Ambion, Austin, TX). SiRNA transcriptional activity was determined by transfecting MCF-7 cells (∼2.0×10^∧^5/well)(50–100 nM siRNAs, Lipofectamine 2000 and/or RNAiMAX, Invitrogen, Carlsbad, CA). The cultures were collected 48 hrs later, counted to determine viability, and mRNA isolated (RNeasy Mini Kit and Qiacube, Qiagen, Valencia, CA). Cellular mRNA was then Dnase treated (Ambion Turbo DNA-free, Austin, TX) and ∼25 ng of cellular mRNA converted to cDNA using either a specific primer (Table S1), or the polydTT primer (iScript cDNA Synthesis kit, BioRad, Hercules, CA). Quantitative RT-PCR was performed and p21 expression determined relative to GAPDH expression with p21qRTPCRFor(Set2), p21qRTPCRRev(Set2), or for E-cadherin using Ecad ForqPCR and Ecad RevqPCR and GAPDHF and GAPDHR primers used (Table S1) as described in [6].

### Dot Blot Analysis of p21 Antisense Expression

MCF-7 cells ∼4×10^∧^6 were transfected with control or siBx332409 (50 nM) and 48 hrs later RNA isolated. A total of 500 ng of cellular RNA was heated to 95°C (10 min), and flash frozen in dry ice for (∼5 min) and blotted onto a prewashed (hybridization buffer [3]) nitrocellulose membrane and cross-linked. The membranes were then exposed to a p21 Bx332409 specific 5′ biotin tagged probe 5′-ACT AAC GTT GAG CCC CTG GAG GCA CTG AAG TGC TTA GTG TAC TTG GAG TAT TGG GGT CTG ACC CCA AAC ACC TTC CAG CTC CTG TAA CAT AC T GGC CTG GAC TGT TTT-3′, which was heated to 95°C (10 min) and flash frozen in dry ice for (∼5 min). The blot and probe were incubated overnight at 37°C in hybridization buffer, washed and then exposed using the Ambion BrightStar BioDetect (Ambion, Austin TX) according to manufactures described procedures.

### Characterizing Bidirectional Transcription

To determine the expression level of the bidirectional transcripts directional RT-PCR was performed. To determine sense directional transcription (relative to the orientation of mRNA production), specifically in the mRNA or coding region of p21 or E-cadherin, the p21pRNA sense primer (Table S1) or E-cadherin RevRace was used for cDNA conversion. Whereas to determine the antisense specific transcription levels (specifically in the coding region of p21 or E-cadherin) the p21RNAantisense or E-cadherin ForRace primers (Table S1) were used for the conversion of mRNA to cDNA as described [13]. When the promoter RNA (pRNA) levels were assessed and inverse situation applied. To characterize the sense directional transcription (relative to the orientation of mRNA production), the p21pRNA Antisense primer (Table S1) was used for cDNA conversion. Whereas to determine the antisense specific transcription levels (specifically in the coding region of p21) the p21RNA Sense primer (Table S1) was used for the conversion of mRNA to cDNA. The converted cDNA was then assessed relative to dTT primed (SuperscriptIII Supermix, Invitrogen, Carlsbad, CA) cDNA using qRT-PCR and primer set 1 (pRNA specific) or 2 (mRNA specific) (Table S1).

### Suppression of Ago-2 Expression

Roughly 5×10^5^ MCF-7 cells were transfected in triplicate with the respective siRNA (Table S1) (100 nM siRNAs, Lipofectamine 2000 and/or RNAiMAX, Invitrogen) and 4 hours later transfected again with a plasmid containing an Ago-2 specific shRNA (500 ng/Lipofectamine 2000). The pENTER/H1/TO-Ago-2 plasmid containing an shRNA targeted to Ago-2 [23] was cloned into the BLOCK-iT Inducible H1 RNAi Entry Vector Kit (Invitrogen, Carlsbad, CA) according to the manufacture described protocol and confirmed by sequence analysis. Forty-eight hours following the last transfection the cultures were collected and p21 expression determined by qRT-PCR analysis as described previously.

### Suppression of Ago-1 Expression

Roughly 5×10^5^ MCF-7 cells were transfected in triplicate with the either control pBSK+ or pSH-Ago-1, an shRNA expressing plasmid (BLOCK-iT, Invitrogen)(∼200 ng plasmid/2×10^∧^5 cells, Lipofectamine 2000) targeted to a previously determined susceptible loci in Ago-1 [23]. Twenty-four hours later the cultures were collected, pooled together, and mRNA assessed by qRT-PCR for Ago-1 expression standardized to GAPDH.

### ChIP Assay

MCF-7 cells (4×10^6^/10 cm plate) were transfected with either p21-322, p21-Bx332409, p21si52, p21si130, p21si858, or the control siRNA R854 (Lipofectamine 2000, 1∶3 Vol∶Vol siRNA (100 nM) to Lipofectamine). Forty-eight hours later the cultures were collected and ChIP assay performed as described [7] using anti-acetyl-Histone 3 (Lys 14)(Upstate, Lake Placid, NY), anti-dimethyl-Histone H3 (Lys4)(Upstate, Lake Placid, NY), anti-di-methyl-Histone 3 (Lys 9)(Upstate, Lake Placid, NY), anti-Tri-methyl-Histone H3 (Lys 27)(Cell Signaling, Boston, MA), or Anti-Ago-1 (Upstate, Lake Placid, NY or Santa Cruz Biotechnology) and primer sets 1, 2, or 3 (Table S1).

### Nuclear Run-On

MCF-7 cells (∼2×10^6^) were transfected in duplicate with siRNAs (p21-322, Bx332409, si-p21-858, or the control CCR5, 50 nM, Lipofectamine 2000). Cells were collected 24 hours post transfection and the nuclear run-on assay was performed according to previously published methods [3],[24] with slight modifications. Biotin labeled RNA was isolated using Streptavidin-coupled Dynabeads (Invitrogen, Carlsbad, CA). Isolate RNA was not eluted from the beads but rather subject to RT-PCR using iScript cDNA Synthesis Kit (BioRad, Hercules, CA) while still linked to the Dynabeads. Directional RT reactions were carried out by the addition of specific oligonucleotides, capable of either sense or antisense specific cDNA conversion (Table S1), directly to the bead mixture. Three independent RT-PCR reactions were carried out for each sample. After the RT-PCR step, samples were centrifuged to pellet the Dynabeads, heated, and the cDNA-containing supernatant was removed. The recovered cDNA was then subject to qRT-PCR to assay the level of luciferase expression relative to GAPDH. A dot blot was also used to measure the respective nuclear run-on following previously established procedures [3]. The relative dot intensity was determined using ImageJ imaging software and treatment samples standardized to GAPDH.

## Supporting Information

Figure S1 Gene activating siRNA p21-322 is predicted to target the p21 antisense transcript EST Bx332409. (A) The p21-322 antisense EST Bx332409 target loci is shown along with the putative target loci in the respective transcript. The siRNA si-Bx332409 was generated to specifically target the EST Bx332409 transcript at the same region where p21-322 is predicted to target the p21 antisense loci (∼bp150 of EST Bx332409). (B) Treatment of MCF-7 cells with siBx332409 results in a reduction in p21 antisense Bx332409 expression. A dot blot on single treated cultures relative to the control is shown with the respective Image J analysis, standard deviations and P values from a paired T-test.
(2.83 MB TIF)

Figure S2 Predicted off-target siRNA binding sites in E-cadherin. (A) Both E-cadherin 640 [5] and miR-373 [11], shown to modulate transcriptional gene activation, can bind E-Cadherin in the coding region or where putative E-cadherin specific antisense RNAs would be predicted to overlap. Several ESTs have been reported and can, based on computational predictions using the program Amplify, bind either E-cadherin 640 and/or miR-373 [14]. (B) E-cadherin 640 can reverse transcribe E-cadherin mRNA. Primers containing sequence homology for E-cadherin 640 antisense (sense/mRNA specific) were used to reverse transcribe total MCF-7 RNA followed by E-cadherin specific PCR. (C) Antisense miR373 can reverse transcribe E-cadherin mRNA. The miR373 antisense primer (sense/mRNA specific) was used to reverse transcribe total MCF-7 RNA which was followed by E-cadherin specific PCR. (D) Cultures treated with Ecad640 or miR373 exhibit increased E-cadherin sense/mRNA expression along with reduced antisense E-cadherin expression relative to untreated cells. MCF-7 cells were transfected with either miR373 or E-cadherin and assayed 48 hrs later by directional RT for E-cadherin expression (using E-cad qPCR primers, Table S1).
(4.24 MB TIF)

Figure S3 Suppression of p21 mRNA expression. MCF-7 cells were transfected with various siRNAs targeted to the p21 sense (mRNA) transcript. Forty-eight hours following transfection the cultures were assessed for p21 mRNA expression relative to GAPDH. The averages from triplicate treated cultures are shown with the standard errors of the mean and P values from a paired T-test.
(2.17 MB TIF)

Figure S4 Suppression of Ago-1. The Ago-1 specific shRNA (pENTR/H1/TO-Ago-1) previously shown to suppress Ago-1 expression [24] is effective at suppressing Ago-1 mRNA expression in MCF-7 cells. The averages with the standard deviations and P values from paired T-test are shown from triplicate measurements of the triplicate treated pooled samples.
(1.73 MB TIF)

Table S1 Oligonucleotide primers used in the current study.
(0.06 MB DOC)

Table S2 p21 sense and antisense expression relative to cell numbers.
(0.04 MB DOC)
