# Supplementary figures and images for: Bidirectional Transcription Directs Both Transcriptional Gene Activation and Suppression in Human Cells
Source: PLoS Genet. 2008 Nov 14;4(11):e1000258. doi: 10.1371/journal.pgen.1000258 (PMC2576438; doi:10.1371/journal.pgen.1000258)

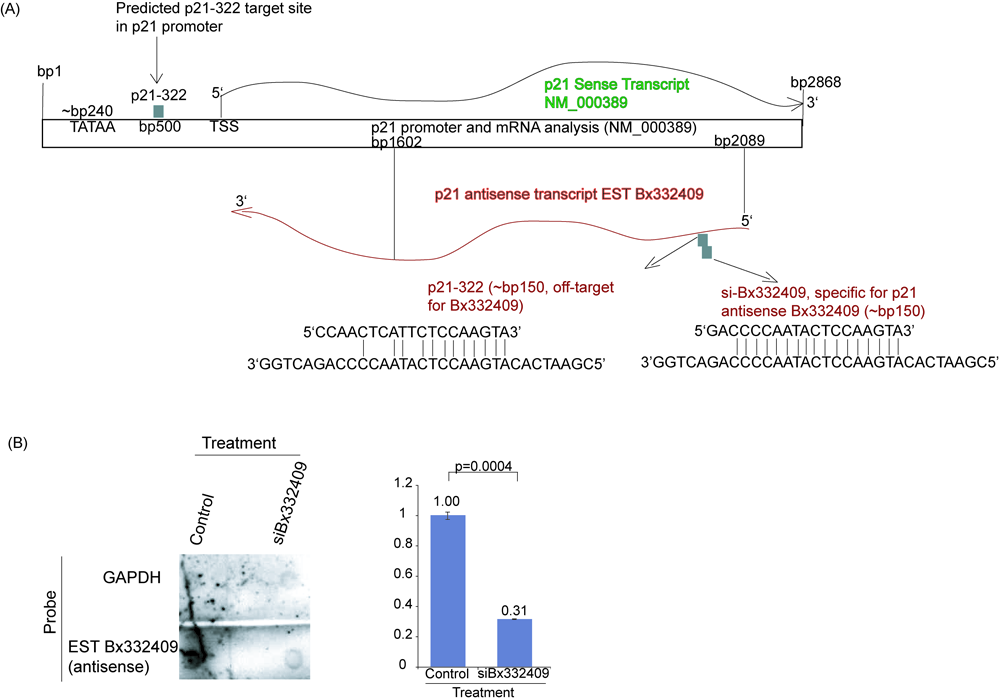

Supplement: Figure S1 — Gene activating siRNA p21-322 is predicted to target the p21 antisense transcript EST Bx332409. (A) The p21-322 antisense EST Bx332409 target loci is shown along with the putative target loci in the respective transcript. The siRNA si-Bx332409 was generated to specifically target the EST Bx332409 transcript at the same region where p21-322 is predicted to target the p21 antisense loci (∼bp150 of EST Bx332409). (B) Treatment of MCF-7 cells with siBx332409 results in a reduction in p21 antisense Bx332409 expression. A dot blot on single treated cultures relative to the control is shown with the respective Image J analysis, standard deviations and P values from a paired T-test. (2.83 MB TIF) [file pgen.1000258.s001.tif]

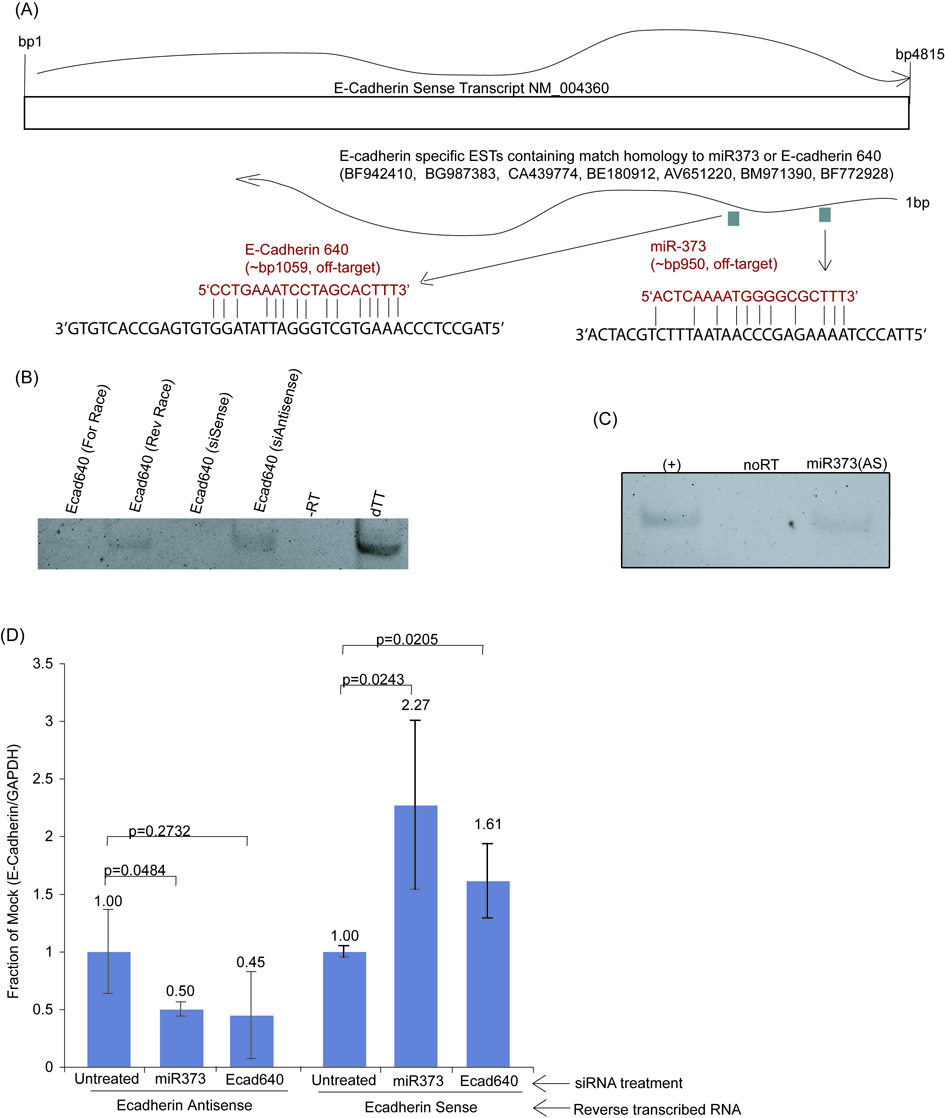

Supplement: Figure S2 — Predicted off-target siRNA binding sites in E-cadherin. (A) Both E-cadherin 640 [5] and miR-373 [11], shown to modulate transcriptional gene activation, can bind E-Cadherin in the coding region or where putative E-cadherin specific antisense RNAs would be predicted to overlap. Several ESTs have been reported and can, based on computational predictions using the program Amplify, bind either E-cadherin 640 and/or miR-373 [14]. (B) E-cadherin 640 can reverse transcribe E-cadherin mRNA. Primers containing sequence homology for E-cadherin 640 antisense (sense/mRNA specific) were used to reverse transcribe total MCF-7 RNA followed by E-cadherin specific PCR. (C) Antisense miR373 can reverse transcribe E-cadherin mRNA. The miR373 antisense primer (sense/mRNA specific) was used to reverse transcribe total MCF-7 RNA which was followed by E-cadherin specific PCR. (D) Cultures treated with Ecad640 or miR373 exhibit increased E-cadherin sense/mRNA expression along with reduced antisense E-cadherin expression relative to untreated cells. MCF-7 cells were transfected with either miR373 or E-cadherin and assayed 48 hrs later by directional RT for E-cadherin expression (using E-cad qPCR primers, Table S1). (4.24 MB TIF) [file pgen.1000258.s002.tif]

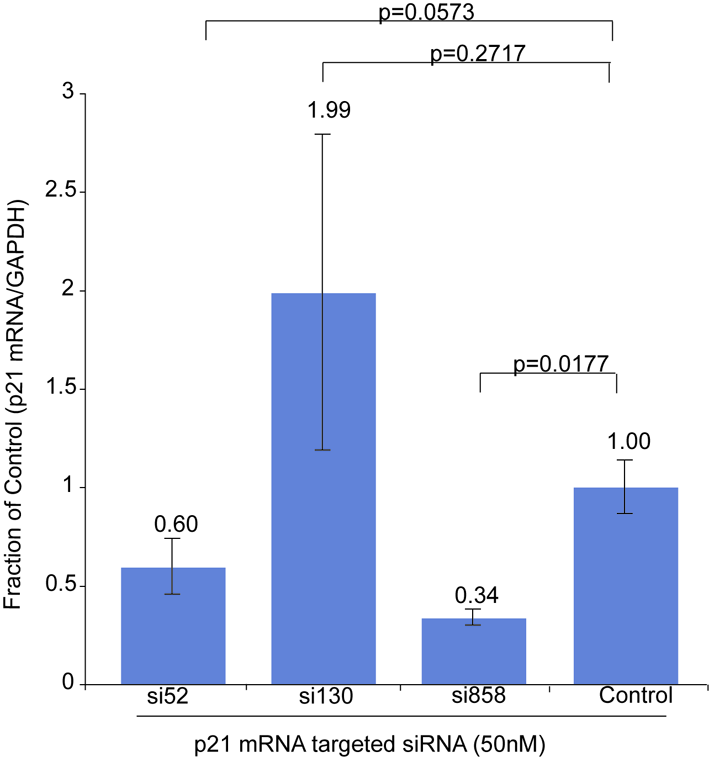

Supplement: Figure S3 — Suppression of p21 mRNA expression. MCF-7 cells were transfected with various siRNAs targeted to the p21 sense (mRNA) transcript. Forty-eight hours following transfection the cultures were assessed for p21 mRNA expression relative to GAPDH. The averages from triplicate treated cultures are shown with the standard errors of the mean and P values from a paired T-test. (2.17 MB TIF) [file pgen.1000258.s003.tif]

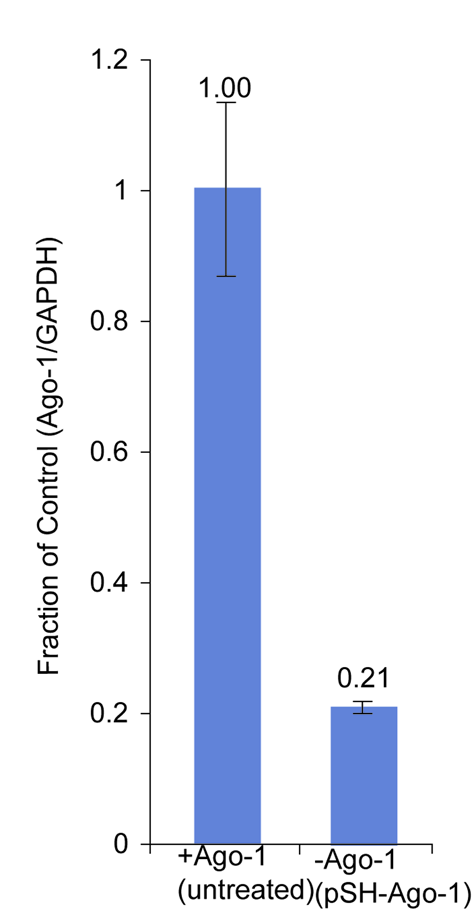

Supplement: Figure S4 — Suppression of Ago-1. The Ago-1 specific shRNA (pENTR/H1/TO-Ago-1) previously shown to suppress Ago-1 expression [24] is effective at suppressing Ago-1 mRNA expression in MCF-7 cells. The averages with the standard deviations and P values from paired T-test are shown from triplicate measurements of the triplicate treated pooled samples. (1.73 MB TIF) [file pgen.1000258.s004.tif]
