# Supplementary material for: Bidirectional Transcription Directs Both Transcriptional Gene Activation and Suppression in Human Cells
Source: PLoS Genet. 2008 Nov 14;4(11):e1000258. doi: 10.1371/journal.pgen.1000258 (PMC2576438; doi:10.1371/journal.pgen.1000258)
Supplement: Table S1 — Oligonucleotide primers used in the current study. (0.06 MB DOC) [file pgen.1000258.s005.doc]

**Table S1** Oligonucleotide primers used in the current study. Only the siRNA target loci are also shown (*) and 5’ linked bio is represented as (Bio-)

| **Oligonucleotide/siRNA** | **Sequence (5’-3’)** |
| --- | --- |
| 5’p21_Prna(Set1) | AAAGGGGGCTCATTCTAAC |
| 3’p21_Prna (Set1) | TTCCTCGCCTGCGTTGG |
| P21For (qRTPCR,Set2) | TTAGCAGCGGAACAAGGAGTCAGA |
| P21Revq(qRTPCR,Set2) | ACACTAAGCACTTCAGTGCCTCCA |
| P21BxCHIPFor(Set3) | GTGGAGAAACGGGAACCAGG |
| P21BxCHIPRev(Set3) | TGGAGGCACTGAAGTGC |
| GAPDH fwd | CGCTGAGTACGTCGTGGAGTC |
| GAPDH rev | GCAGGAGGCATTGCTGATGA |
| *P21-322-(promoter targeted) | CCAACTCATTCTCCAAGTA |
| *P21-Bx332409 (antisense targeted) | AAATGAACCTCATAACCCCAG |
| *Control (CCR5,R854) | AATTCTTTGGCCTGAATAATT |
| p21-322Sense (directional RTPCR) | CCAACTCATTCTCCAAGTA |
| p21-322Antisense (directional RTPCR) | TACTTGGAGAATGAGTTGG |
| *p21si-858(mRNA targeted) | AAATACTATTTAAAGCCTCCT |
| *p21 si-52(mRNA targeted) | AAGTCAGTTCCTTGTGGAGCC |
| *p21 si130(mRNA targeted) | AACCGGCTGGGGATGTCCGTC |
| miR373 ForRace | ACTCAAAATGGGGCGCTTT |
| miR373 RevRace | AAAGCGCCCCATTTTGAGT |
| Ecad ForRace | CCTGAAATCCTAGCACTTT |
| Ecad RevRace | AAAGTGCTAGGATTTCAGG |
| Ecad ForqPCR | AAGAAGCTGGCTGACATGTACGGA |
| Ecad RevqPCR | CCACCAGCAACGTGATTTCTGCAT |
| Ecad640 (siSense) | AAAAAGTGCTAGGATTTCAGGCCTGTCTC |
| Ecad640 (siAntisense) | AACCTGAAATCCTAGCACTTTCCTGTCTC |
| *miR373 (E-cadherin promoter targeted) | AACTCAAAATGGGGCGCTTTCC |
| *Ecad640 (E-cadherin promoter targeted) | AACCTGAAATCCTAGCACTTT |
