# Supplementary material for: Bidirectional Transcription Directs Both Transcriptional Gene Activation and Suppression in Human Cells
Source: PLoS Genet. 2008 Nov 14;4(11):e1000258. doi: 10.1371/journal.pgen.1000258 (PMC2576438; doi:10.1371/journal.pgen.1000258)
Supplement: Table S2 — p21 sense and antisense expression relative to cell numbers. (0.04 MB DOC) [file pgen.1000258.s006.doc]

**Table S2** p21 sense and antisense expression relative to cell numbers. Directional RT PCR was performed and relative copies of either p21 sense or antisense were standardized to GAPDH and cell numbers. The averages are shown from triplicate transfected cultures with the standard deviations.

| **Treatment** | **p21 (Sense/Cell)** | **p21 (antisense/Cell)** | **p21 (Antisense/Sense)/ Cell** |
| --- | --- | --- | --- |
| p21-322 | 145.2 +/-13.03 | 17.81 +/-8.28 | 0.12 +/- 0.049 |
| siBx332409 | 1775.74 +/-650.72 | 28.47 +/-24.68 | 0.01 +/- 0.018 |
| Control | 25.15 +/-12.52 | 20.26 +/-15.59 | 0.33 +/- 0.01 |
| Untreated | 5.95 +/-6.17 | 2.68 +/- 0.397 | 0.82 +/- 0.54 |
